# Hematological and blood biochemistry parameters as prognostic indicators of survival in canine multicentric lymphoma treated with COP and L-COP protocols

**DOI:** 10.14202/vetworld.2024.344-355

**Published:** 2024-02-08

**Authors:** Somchin Sutthigran, Phasamon Saisawart, Patharakrit Teewasutrakul, Sirintra Sirivisoot, Chutimon Thanaboonnipat, Anudep Rungsipipat, Nan Choisunirachon

**Affiliations:** 1 Department of Surgery, Faculty of Veterinary Science, Chulalongkorn University, Bangkok 10330, Thailand; 2 Small Animal Teaching Hospital, Faculty of Veterinary Science, Chulalongkorn University, Henri Dunant Rd., Pathumwan, Bangkok 10330, Thailand; 3 Center of Excellence for Companion Animal Cancer, Department of Veterinary Pathology, Faculty of Veterinary Science, Chulalongkorn University, Henri Dunant Rd., Pathumwan Bangkok10330, Thailand

**Keywords:** anti-cancer, chemotherapy, dogs, hypoalbuminemia, multicentric lymphoma, prognosis, retrospective study, survival outcomes

## Abstract

**Background and Aim:**

Hematological and blood chemistry parameters are crucial for evaluating and monitoring canine multicentric lymphoma during chemotherapy. Pre-treatment hematological and blood chemistry parameters can be used as prognostic survival outcomes for this disease. Therefore, this study aimed to investigate the effect of hematological and blood chemistry parameters pre-treatment and 4 weeks post-treatment on the survival outcomes of dogs treated with either a combination of cyclophosphamide, vincristine, and prednisolone (COP) or a combination of COP with L-asparaginase (L-COP) protocols.

**Materials and Methods:**

We conducted a retrospective study. Medical records and hematological and blood chemistry parameters of 41 dogs with multicentric lymphoma treated with L-COP (n = 26) and the COP protocols (n = 15) were obtained from the hospital information system. Most cases were classified as high-grade lymphoma based on the Kiel cytological classification. The effects of hematological and blood chemistry parameters on survival outcomes were investigated using the Cox proportional hazard regression model. The median survival time (MST) for each hematological and blood chemistry parameter affecting survival outcome was established and compared using the Kaplan–Meier product limit method with the log-rank test.

**Results:**

Dogs with high-grade multicentric lymphoma that were treated with the COP protocol and had monocytosis at pre-treatment had a significantly shorter MST than dogs with normal monocyte counts (p = 0.033). In addition, dogs with azotemia, both pre-treatment and 4 weeks post-treatment, had a significantly shorter MST than dogs with normal serum creatinine levels (p = 0.012). Dogs with high-grade multicentric lymphoma treated with the L-COP protocol who had hypoalbuminemia (serum albumin concentration <2.5 mg/dL) at both pre-treatment and 4 weeks post-treatment had a significantly shorter MST than dogs with normal serum albumin levels (p < 0.001). Furthermore, dogs with leukocytosis at 4 weeks post-treatment had a significantly shorter MST than those with a normal total white blood cell count (p = 0.024).

**Conclusion:**

Serum albumin level can serve as a simple negative prognostic indicator of survival outcomes in dogs with high-grade multicentric lymphoma treated with the L-COP protocol. Dogs with hypoalbuminemia pre-treatment and 4 weeks post-treatment tended to have a shorter MST than those with normal serum albumin concentrations.

## Introduction

Canine lymphoma is a common hematopoietic neoplasm that originates from lymphocytes and accounts for 83% of all canine hematopoietic neoplasms. It usually arises in lymphoid tissues, particularly in the lymph nodes. More than 80% of this disease presents in a multicentric form and is characterized by peripheral lymphadenopathy [1]. Nevertheless, it can also develop in other lymphoid tissues, such as the spleen and bone marrow [1]. The annual incidence rate of this disease is reported to be between 21 and 120/100,000 dogs [1–3], and the incidence rate tends to increase annually, similar to that in humans [4]. In addition, canine lymphoma can be classified into subtypes using the Revised European American Lymphoma/World Health Organization (REAL/WHO) system. This system comprises anatomical, morphological, and immunophenotypic criteria (B-cell and T-cell immunophenotypes) [1]. The most common subtypes are diffuse large B-cell lymphoma, peripheral T-cell lymphoma not otherwise specified, T-zone lymphoma, T-lymphoblastic lymphoma, and marginal-zone lymphoma [5].

Chemotherapy is the treatment of choice for canine multicentric lymphoma. Although several chemotherapeutic protocols have been used, the most commonly used is the CHOP-based protocol which consists of cyclophosphamide, doxorubicin, vincristine, and prednisolone owing to its high response rate [1, 6]. However, doxorubicin has been reported to be associated with several adverse effects, such as myelosuppression, gastrointestinal symptoms, and cardiac toxicity [7, 8]. The cyclophosphamide, vincristine, and prednisolone (COP) protocol or its combination of L-asparaginase (L-COP) has been reported to be an effective treatment protocol with fewer adverse effects [9, 10]. Considering the therapeutic strategy for dogs with multicentric lymphoma, hematological and blood chemistry parameters are crucial for evaluating patient status, making treatment decisions, and monitoring disease progression and adverse chemotherapeutic effects [1]. Moreover, hematological and blood chemistry parameters during the pre-treatment period have been proven to be valuable prognostic factors for survival outcomes in canine lymphomas, such as hematocrit [11], lymphocyte-to-monocyte ratio (LMR) [12], platelet count [11, 13], globulin concentration [13], and albumin concentration [14, 15].

To the best of our knowledge, information regarding the effects of post-treatment hematological and blood chemistry parameters on survival outcomes remains lacking. Moreover, the predictive value of hematological and blood chemistry parameters associated with survival outcomes in dogs with multicentric lymphoma treated with the COP or L-COP protocols has not been elucidated.

Therefore, this study aimed to investigate the association between hematological and blood chemistry parameters at pre-treatment and 4 weeks post-treatment and survival outcomes in dogs with multicentric lymphoma treated with either the COP or L-COP protocols. In addition, this study aimed to establish a cutoff value for hematological and blood chemistry parameters that affect survival outcomes.

## Materials and Methods

### Ethical approval

This study was performed in accordance with the institutional guidelines and regulations, followed the Animal Research: Reporting of *In Vivo* Experiments (ARRIVE) guidelines and was approved by the hospital’s institutional review board (approval number S393/2565), Faculty of Veterinary Science, Chulalongkorn University.

### Study period and location

The study included medical records and laboratory results, including hematology and serum biochemistry of dogs with multicentric lymphoma that were presented to the Small Animal Hospital, Faculty of Veterinary Science, Chulalongkorn University from January 2018 to December 2021.

### Case selection

This study was designed as a cross-sectional retrospective study based on medical records obtained from the hospital information system. The inclusion criteria included medical records of dogs diagnosed with multicentric lymphoma, as determined by cytologic results from fine needle aspiration or histopathological results from lymph node biopsy. These dogs were treated with either the combination of cyclophosphamide (250 mg/m^2^ orally divided over 2 consecutive days at the 2^nd^ week), vincristine (0.7 mg/m^2^ intravenous injection at the 1^st^ week), and prednisolone (2 mg/kg orally daily at the 1^st^ week and tapering for 4 weeks to 0.5 mg/kg orally daily) (COP) or the combination of COP with L-asparaginase (400 units/kg subcutaneous injection once at the 1^st^ week) (L-COP), followed by the continuation of COP every 3 weeks at the same dosage for at 1 year as maintenance. The treatment protocols were randomly assigned based on the oncologist’s decision, considering the patient’s condition and the owner’s financial situation. The exclusion criteria included medical records of dogs diagnosed with other forms of lymphoma, dogs treated with different chemotherapeutic protocols, dogs with adjusted or delayed treatment, and dogs previously treated with chemotherapy or steroids.

### Data collection

Data, including age, gender, breed, body weight, survival time (ST), and hematological and blood chemistry data, were collected from medical records. Multicentric lymphoma was diagnosed by veterinary pathologists through cytological examination. Fine needle aspiration of the affected lymph nodes was performed, followed by fixation with methanol and Giemsa staining. In addition, a peripheral blood smear was examined, and the substage, which was determined by the presence or absence of systemic signs, was recorded. Finally, the clinical staging was established based on the WHO staging system and recorded [16] (Supplementary data). Moreover, the Kiel cytological classification was determined based on the cytological results obtained from the affected lymph nodes. Hematology was performed using an auto hematology analyzer (model BC-5000Vet; Shenzhen Mindray Animal Medical Technology, China), and blood chemistry was performed using a chemistry analyzer (model BS-800; Shenzhen Mindray Animal Medical Technology, China). Hematological and blood chemistry data were collected pre-treatment and 4 weeks post-treatment, following the standardized clinical re-evaluation recommended by the Veterinary Cooperative Oncology Group guideline [17]. The collected data consisted of the complete blood count, liver enzymes (alanine aminotransferase [ALT], alkaline phosphatase [ALP]), kidney function test (blood urea nitrogen [BUN], creatinine, BUN-to-creatinine ratio), total protein, globulin, albumin concentration, and albumin-to-globulin ratio (AGR). The peripheral blood ratios, such as neutrophils-to-lymphocyte ratio, LMR, platelets-to-neutrophils-ratio, and platelets-to-lymphocyte ratio (PLR), were calculated and recorded. The levels of each hematological and blood chemistry parameter and peripheral blood ratio were determined based on the normal reference values of the concerned laboratory.

### Statistical analysis

Statistical analyses were performed using a commercial software program (Statistical Package for the Social Sciences Statistics software for MacOS version 29, IBM Corp., New York, USA). Statistical analysis was selected to evaluate the data, utilizing the standard method [18]. Clinical data were presented as descriptive data. Continuous data were assessed for normal distribution using the Shapiro–Wilk normality test and were reported as mean ± standard deviation for normally distributed data and median with range for non-normally distributed data. Categorical data, including the WHO stages and substages, were expressed as numbers and percentages. The paired sample t-test was used to determine the differences in hematological parameters between pre-treatment and 4 weeks post-treatment. A receiver operating characteristic (ROC) curve and area under the curve (AUC) were established to determine the optimal cutoff values of hematological parameters affecting overall survival at 180 and 365 days. The Cox proportional hazard regression model was used to evaluate the effect of hematological parameters on overall survival at 180 and 365 days. ST curves were constructed using the Kaplan–Meier product limit method and compared using the log-rank test for each hematological parameter. Differences were considered statistically significant at p < 0.05.

## Results

### Clinical demographic data

The medical records of 41 dogs with multicentric lymphoma were included in the study. The diagnosis was established through cytological results, as opposed to histopathological examination, owing to certain circumstances, such as the presence of thrombocytopenia, posing a high risk of bleeding disorder during the initial presentation. Some cases involving aggressive canine patients and those with high American Society of Anesthesiologists scores, sedation, and general anesthetic procedures were considered contraindications for biopsy. Twenty-six (63.41%) dogs were treated with the L-COP protocol, whereas 15 (36.59%) were treated with the COP protocol. According to the Kiel cytological classification, 25 of the 26 dogs treated with the L-COP protocol were classified as having high-grade lymphoma, whereas one dog was classified as having low-grade lymphoma. Similarly, 13 of 15 dogs treated with COP protocol were classified as having high-grade lymphoma, and one dog was classified as having low-grade lymphoma. However, one dog treated using the COP protocol could not be classified because of insufficient cytological results.

Regarding dogs with multicentric lymphoma treated with the COP protocol, the enrolled dogs included mixed-breed dogs (n = 4), Shih Tzu (n = 4), and one each of American Bully, Bulldog, Beagle, French Bulldog, Rottweiler, Scottish Terrier, and Yorkshire Terrier, whereas dogs with multicentric lymphoma treated with the L-COP protocol included mixed-breed dogs (n = 5), Jack Russel Terrier (n = 4), Beagle (n = 1), Chihuahua (n = 2), French Bulldog (n = 2), Golden Retriever (n = 2), and one each of Bulldog, Cocker Spaniel, Finnish Spitz, Labrador Retriever, Pitbull, Pomeranian, Poodle, Shih Tzu, Siberian Husky, and Yorkshire Terrier. Two dogs treated with the L-COP protocol and one dog treated with the COP protocol died within 4 weeks of the initial treatment. The clinical demographic data of the enrolled dogs are presented in Table-1. Of the 41 enrolled dogs, seven were classified as substage b, which is defined as the presence of systemic signs. The most common systemic signs observed were anorexia (100%, 7/7) and weight loss (100%, 7/7). The hematological parameters, including the peripheral blood ratio of the enrolled dogs at pre-treatment and 4 weeks post-treatment (Supplementary data). Statistically significant differences in hematological and blood chemistry parameters in dogs with multicentric lymphoma treated with the COP protocol were detected between the pre-treatment and 4 weeks post-treatment periods, especially lymphocyte count (p = 0.022), monocyte count (p = 0.039), platelet count (p = 0.009), ALP (p = 0.016), BUN (p = 0.017), creatinine (p < 0.001), globulin (p = 0.001), albumin (p < 0.001), PLR (p = 0.013), and AGR (p < 0.001). Nonetheless, the hematological parameters of dogs treated with the L-COP protocol were not significantly different between the pre-treatment and 4 weeks post-treatment periods.

**Table-1:** Clinical demographic information of dogs treated with L-COP protocol and COP protocol.

Characteristics	Dogs treated with L-COP (n = 26)	Dogs treated with COP (n = 15)
Age (years)		
Median (range)	10 (4–15)	10 (3–15)
Gender n, %		
Male	16 (61.54)	11 (73.33)
Female	10 (38.46)	4 (26.67)
Body weight (kg)		
Median (range)	8.75 (2.50–37.90)	12.33 (4.00–28.00)
WHO stage and substage n, %		
IIIa	4 (15.38)	5 (33.33)
Ib	0 (0.00)	0 (0.00)
IVa	13 (50.00)	8 (53.33)
IVb	4 (15.38)	2 (13.34)
Va	4 (15.38)	0 (0.00)
Vb	1 (3.86)	0 (0.00)

L-COP=Cyclophosphamide, vincristine, and prednisolone with L-asparaginase, COP=Cyclophosphamide, vincristine, and prednisolone

### Survival

The median ST (MST) of dogs treated with the COP protocol was 371 days (95% confidence interval [CI], 233.37–976.75) and for those treated with the L-COP protocol was 235 days (95%CI, 164.59–351.07). The survival rates of dogs treated with the COP protocol were 60% (9/15) and 53.33% (8/15) at 180 and 365 days, respectively. In contrast, the survival rates of dogs using the L-COP protocol were 57.69% (15/26) and 19.23% (5/26) at 180 days and 365 days, respectively.

**Figure-1:**
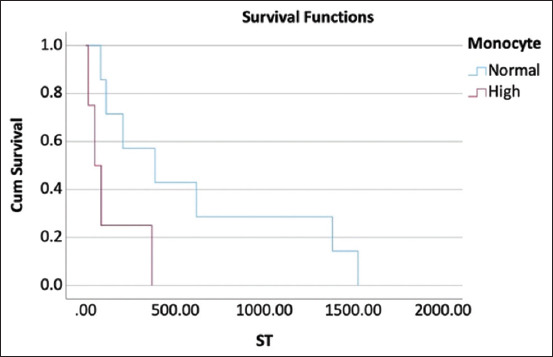
Kaplan–Meier curve represents median survival time of dogs with monocytosis at pre-treatment and those with normal monocyte count that were treated with cyclophosphamide, vincristine, and prednisolone protocol were 51 days and 388 days, respectively, at p = 0.033.

**Figure-2:**
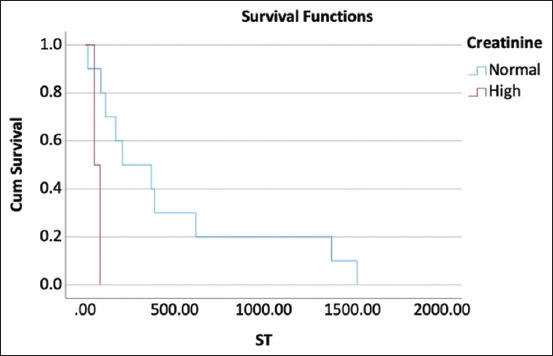
Kaplan–Meier curve represents median survival time of dogs with high creatinine concentration at pre-treatment and those with normal creatinine concentration that were treated with cyclophosphamide, vincristine, and prednisolone protocol were 51 days and 208 days, respectively, at p = 0.012.

In dogs treated with the COP protocol, univariate analyses showed that pre-treatment monocytosis significantly affected overall survival at 180 days (Hazard ratio [HR] = 6.515; p = 0.033). In addition, azotemia at pre-treatment significantly influenced overall survival at 180 days (HR = 11.916; p = 0.045) and 365 days (HR = 11.916; p = 0.045). However, there was no significant association between the parameters in the multivariate analysis. The results of the univariate and multivariate analyses for each hematological parameter pre-treatment and 4 weeks post-treatment are shown in Tables-2 and 3. Considering monocyte, the MST of dogs with monocytosis pre-treatment was significantly different from that of dogs with normal monocyte count at 51 days (95%CI, 0.00–122.54) versus 388 days (95%CI, 0.00–849.92) (p = 0.033) (Figure-1). Moreover, the MST of dogs with azotemia pre-treatment was significantly different from that of dogs with normal creatinine concentrations (51 days vs. 208 days, p = 0.012) (Figure-2). Univariate analysis revealed that substage significantly impacted overall survival at 180 and 365 days (HR = 11.916; p = 0.045). There was a significant difference between the MST of dogs presenting with substages a and b (388 days vs. 51 days) (p = 0.003). However, the stages had no significant effect on overall survival at 180 and 365 days (p = 0.241 and p = 0.221, respectively).

**Table-2:** Cox proportional analysis for hematological abnormalities at pre-treatment to overall survival at 180 and 365 days in 15 dogs treated with COP protocol.

Abnormality	n (%)	Univariate							

		180 days				365 days			
	
		HR		CI95%	p-value	HR		CI95%	p-value
Anemia	7 (46.67)	1.073		0.237–4.863	0.927	1.903		0.368–9.846	0.443
Leukocytosis	1 (7.00)	2.181		0.490–9.709	0.306	2.244		0.506–9.961	0.288
Neutrophilia	0 (0.00)	-		-	-	-		-	-
Eosinophilia	0 (0.00)	-		-	-	-		-	-
Lymphocytosis	0 (0.00)	-		-	-	-		-	-
Monocytosis	4 (26.67)	6.515		1.160–36.601	0.033	3.298		0.643–16.920	0.153
Thrombocytopenia	2 (13.33)	3.163		0.573–17.466	0.187	3.163		0.573–17.466	0.187
High ALT	7 (46.67)	6.096		0.728–51.074	0.096	2.643		0.503–13.895	0.251
High ALP	8 (53.33)	1.183		0.226–6.197	0.843	0.683		0.152–3.074	0.619
High BUN	4 (26.67)	1.957		0.432–8.867	0.384	2.014		0.442–9.170	0.366
High creatinine	2 (13.33)	11.916		1.053–134.903	0.045	11.916		1.053–134.903	0.045
Hypoproteinemia	0 (0.00)	-		-	-	-		-	-
Hyperglobulinemia	8 (53.33)	1.128		0.217–5.862	0.886	1.078		0.208–5.576	0.929
Hypoalbuminemia	0 (0.00)	-		-	-	-		-	-
NLR	8 (53.33)	0.424		0.077–2.339	0.325	0.826		0.091–7.470	0.865
LMR	9 (60.00)	25.778		0.001–592370.799	0.526	25.031		0.000–2296086.274	0.581
PNR	5 (33.33)	0.154		0.017–1.424	0.099	0.594		0.104–3.386	0.558
PLR	8 (53.33)	0.424		0.077–2.339	0.325	1.127		0.219–5.789	0.886
BCR	7 (46.67)	0.423		0.092–1.950	0.270	1.109		0.220–5.585	0.900
AGR	11 (73.33)	25.044		0.001–518320.200	0.525	27.335		0.002–318371.052	0.489

**Abnormality**	**n (%)**	**Multivariate**							

		**180 days**				**365 days**			
	
		**HR**	**CI95%**		**p-value**	**HR**	**CI95%**		**p-value**

Monocytosis	4 (26.67)	5.741	0.654–50.417		0.115				
High creatinine	2 (13.33)	2.147	0.287–16.041		0.457				

AGR=Albumin-to-globulin ratio, ALP=Alkaline phosphatase, ALT=Alanine aminotransferase, BCR=Blood urea nitrogen-to-creatinine ratio, BUN=Blood urea nitrogen, CI95%=95% confidence interval, HR=Hazard ratio, LMR=Lymphocyte-to-monocyte ratio, NLR=Neutrophil-to-lymphocyte ratio, PLR=Platelet-to-lymphocyte ratio, PNR=Platelet-to-neutrophil ratio, COP=Cyclophosphamide, vincristine, and prednisolone

**Table-3:** Cox proportional analysis for hematological abnormalities at post-treatment at 4 weeks to overall survival at 180 and 365 days in 14 dogs treated with COP protocol.

Abnormality	n (%)	180 days			365 days		
	
		HR	CI95%	p-value	HR	CI95%	p-value
Anemia	8 (57.14)	1.311	0.237–7.239	0.756	1.748	0.209–14.604	0.606
Leukocytosis	2 (14.29)	5.210	0.722–37.595	0.102	5.210	0.722–37.595	0.102
Neutrophilia	2 (14.29)	4.357	0.604–31.442	0.144	4.356	0.604–31.442	0.144
Eosinophilia	1 (7.14)	1.166	0.128–10.602	0.892	1.166	0.128–10.602	0.892
Lymphocytosis	1 (7.14)	0.642	0.069–6.013	0.698	0.033	0.000–185.629	0.439
Monocytosis	2 (14.29)	4.357	0.604–31.442	0.144	4.357	0.064–31.442	0.144
Thrombocytopenia	4 (28.57)	2.415	0.434–13.437	0.314	1.263	0.246–6.493	0.780
High ALT	2 (14.29)	0.321	0.034–3.006	0.319	0.024	0.000–32.266	0.310
High ALP	6 (42.85)	0.126	0.013–1.261	0.078	0.126	0.013–1.261	0.078
High BUN	2 (14.29)	1.023	0.113–9.252	0.984	1.951	0.353–10.780	0.443
High creatinine	2 (14.29)	1.808	0.323–10.114	0.500	0.772	0.089–6.671	0.814
Hypoproteinemia	0 (0.00)	-	-	-	-	-	-
Hyperglobulinemia	5 (35.71)	0.563	0.113–2.819	0.485	0.527	0.104–2.657	0.438
Hypoalbuminemia	0 (0.00)	-	-	-	-	-	-
NLR	6 (42.86)	43.729	0.019–99355.152	0.338	58.525	0.058–58841.806	0.249
LMR	7 (50.00)	1.558	0.166–14.589	0.698	30.264	0.005–170021.239	0.439
PNR	3 (21.43)	0.269	0.031–2.321	0.232	0.594	0.104–3.386	0.558
PLR	4 (28.57)	0.623	0.113–3.440	0.587	1.127	0.219–5.789	0.886
BCR	4 (28.57)	0.610	0.109–3.412	0.574	1.109	0.220–5.585	0.900
AGR	8 (57.14)	29.267	0.003–263021.715	0.467	27.335	0.02–318371.052	0.489

AGR=Albumin-to-globulin ratio, ALP=Alkaline phosphatase, ALT=Alanine aminotransferase, BCR=Blood urea nitrogen-to-creatinine ratio, BUN=Blood urea nitrogen, CI95%=95% confidence interval, HR=Hazard ratio, LMR=Lymphocyte-to-monocyte ratio, NLR=Neutrophil-to-lymphocyte ratio, PLR=Platelet-to-lymphocyte ratio, PNR=Platelet-to-neutrophil ratio, COP=Cyclophosphamide, vincristine, and prednisolone

**Figure-3:**
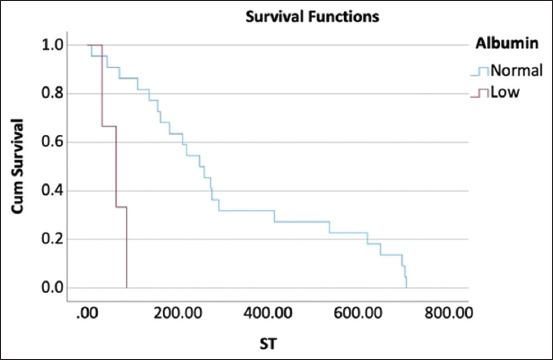
Kaplan–Meier curve represents median survival time of dogs with hypoalbuminemia and dogs with normal serum albumin concentration that was treated with cyclophosphamide, vincristine, and prednisolone with L-asparaginase protocol were 64 days and 249 days, respectively, at p < 0.001.

**Figure-4:**
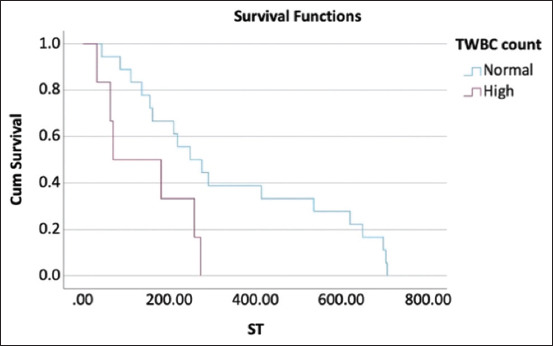
Kaplan–Meier curve represents the median survival time of dogs with leukocytosis and those with normal white blood cell count that were treated with cyclophosphamide, vincristine, and prednisolone with L-asparaginase protocol were 71 days and 249 days, respectively, at p = 0.024. TWBC=Total white blood cells.

For dogs treated with the L-COP protocol, the results of univariate and multivariate analyses in for each hematological and blood chemistry parameter pre-treatment and 4 weeks post-treatment are shown in Tables-4 and 5. Of these parameters, hypoalbuminemia (<2.5 mg/dL) pre-treatment and 4 weeks post-treatment had significantly influenced overall survival at 180 days (HR = 4.018; p = 0.025 and HR = 11.247; p = 0.004, respectively). In addition, hypoalbuminemia at 4 weeks post-treatment affected overall survival at 365 days (HR = 11.247; p = 0.004). The Kaplan–Meier survival curve and log-rank test showed a significant difference between the MST of dogs with hypoalbuminemia and dogs with a normal serum albumin concentration at 64 days (95% CI, 14.39–133.61) versus 249 days (95% CI, 77.75–320.25) (p < 0.001) (Figure-3). Leukocytosis at 4 weeks post-treatment also significantly impacted overall survival at 365 days (HR = 3.123; p = 0.032). The MST of dogs with leukocytosis and dogs with a normal white blood cell count was significantly different at 71 days (95% CI, 0.00–212.63) versus 249 days (95% CI, 132.58–365.42) (p = 0.024) (Figure-4). However, multivariate analysis revealed no significant association between hypoalbuminemia, leukocytosis, and overall survival (Table-6). Considering stage and substage, univariate analysis revealed no significant differences in overall survival at 180 days (p = 0.887 and p = 0.281, respectively) and 365 days (p = 0.176 and p = 0.593, respectively).

**Table-4:** Cox proportional analysis for hematological abnormalities at pre-treatment to overall survival at 180 and 365 days in 26 dogs treated with L-COP protocol.

Abnormality	n (%)	180 days			365 days		
	
		HR	CI95%	p-value	HR	CI95%	p-value
Anemia	15 (57.69)	2.636	0.697–9.975	0.153	1.327	0.532–3.310	0.544
Leukocytosis	11 (42.30)	1.186	0.361–3.891	0.779	1.501	0.605–3.724	0.381
Neutrophilia	8 (30.77)	2.159	0.654–7.129	0.207	1.287	0.487–3.405	0.611
Eosinophilia	4 (15.38)	0.035	0.000–21.898	0.308	0.432	0.100–1.876	0.263
Lymphocytosis	6 (23.08)	0.696	0.150–3.225	0.643	1.279	0.458–3.575	0.639
Monocytosis	8 (30.77)	1.321	0.386–4.518	0.657	1.217	0.461–3.215	0.692
Thrombocytopenia	15 (57.69)	0.844	0.257–2.767	0.779	1.000	0.401–2.492	1.000
High ALT	10 (40.00)	2.738	0.771–9.725	0.119	1.522	0.599–3.868	0.378
High ALP	16 (64.00)	2.534	0.537–11.948	0.240	1.099	0.425–2.843	0.846
High BUN	5 (20.00)	1.099	0.233–5.179	0.905	0.719	0.207–2.500	0.604
High creatinine	1 (4.00)	0.046	0.000–14337.009	0.633	0.904	0.119–6.866	0.923
Hypoproteinemia	10 (40.00)	1.692	0.489–5.861	0.407	1.476	0.579–3.764	0.415
Hyperglobulinemia	4 (16.00)	1.372	0.291–6.480	0.690	0.625	0.143–2.728	0.532
Hypoalbuminemia	8 (30.77)	4.018	1.192–13.540	0.025	2.239	0.785–6.386	0.132
NLR	20 (76.92)	1.436	0.310–6.654	0.643	1.019	0.365–2.843	0.972
LMR	20 (76.92)	1.436	0.310–6.654	0.643	0.782	0.280–2.185	0.639
PNR	5 (19.23)	0.795	0.172–3.686	0.770	0.587	0.170–2.028	0.400
PLR	10 (38.46)	2.245	0.656–7.685	0.198	1.851	0.739–4.636	0.188
BCR	17 (65.38)	6.343	0.801–50.243	0.080	1.763	0.625–4.978	0.284
AGR	18 (69.23)	28.602	0.036–22928.940	0.326	0.705	0.264–1.882	0.485

AGR=Albumin-to-globulin ratio, ALP=Alkaline phosphatase, ALT=Alanine aminotransferase, BCR=Blood urea nitrogen-to-creatinine ratio, BUN=Blood urea nitrogen, CI95%=95% confidence interval, HR=Hazard ratio, LMR=Lymphocyte-to-monocyte ratio, NLR=Neutrophil-to-lymphocyte ratio, PLR=Platelet-to-lymphocyte ratio, PNR=Platelet-to-neutrophil ratio, COP=Cyclophosphamide, vincristine, and prednisolone, L-COP=Cyclophosphamide, vincristine, and prednisolone with L-asparaginase

**Table-5:** Cox proportional analysis for hematological abnormalities at post-treatment at 4 weeks to overall survival at 180 and 365 days in 24 dogs treated with L-COP protocol.

Abnormality	n (%)	180 days						365 days				
	
		HR		CI95%		p-value		HR		CI95%		p-value
Anemia	11 (45.83)	0.619		0.155–2.481		0.499		0.994		0.381–2.590		0.990
Leukocytosis	6 (25.00)	2.123		0.528–8.544		0.289		3.123		1.103–8.841		0.032
Neutrophilia	7 (29.17)	1.631		0.388–6.858		0.504		2.370		0.809–6.949		0.116
Eosinophilia	4 (16.67)	0.596		0.073–4.850		0.629		0.667		0.149–2.984		0.596
Lymphocytosis	1 (4.16)	0.045		0.000–22634.586		0.644		1.953		0.244–15.658		0.529
Monocytosis	4 (16.67)	0.648		0.080–5.281		0.685		2.345		0.701–7.843		0.166
Thrombocytopenia	3 (12.50)	3.875		0.791–18.985		0.095		1.545		0.350–6.815		0.565
High ALT	10 (41.67)	0.623		0.149–2.614		0.518		0.752		0.279–2.026		0.573
High ALP	15 (62.50)	0.675		0.181–2.526		0.560		0.804		0.305–2.117		0.659
High BUN	10 (41.67)	1.198		0.321–4.468		0.788		1.380		0.530–3.593		0.510
High creatinine	3 (12.50)	1.050		0.131–8.416		0.963		1.581		0.447–5.592		0.477
Hypoproteinemia	3 (12.50)	0.380		0.000–84.901		0.405		0.287		0.308–2.181		0.228
Hyperglobulinemia	8 (33.33)	2.410		0.645–9.008		0.191		1.351		0.487–3.744		0.563
Hypoalbuminemia	4 (16.67)	11.247		2.194–57.654		0.004		11.247		2.194–57.654		0.004
NLR	14 (58.33)	2.839		0.341–23.612		0.334		1.619		0.445–5.898		0.465
LMR	11 (45.83)	0.227		0.046–1.133		0.071		0.359		0.121–1.063		0.064
PNR	9 (37.5)	1.189		0.297–4.761		0.807		0.953		0.330–2.755		0.929
PLR	14 (58.33)	1.000		0.194–5.160		1.000		0.580		0.188–1.789		0.343
BCR	18 (75.00)	36.345		0.091–1446.312		0.239		2.247		0.643–7.850		0.204
AGR	18 (75.00)	2.464		0.308–19.719		0.395		1.662		0.471–5.872		0.430

**Abnormality**	**n (%)**	**Multivariate**										

		**180 days**					**365 days**					
	
		**HR**	**CI95%**		**p-value**		**HR**		**CI95%**		**p-value**	

Leukocytosis	6 (25.00)						2.795		0.943–8.285		0.064	
Hypoalbuminemia	3 (12.50)						17.094		2.543–114.887		0.003	

AGR=Albumin-to-globulin ratio, ALP=Alkaline phosphatase, ALT=Alanine aminotransferase, BCR=Blood urea nitrogen-to-creatinine ratio, BUN=Blood urea nitrogen, CI95%=95% confidence interval, HR=Hazard ratio, LMR=Lymphocyte-to-monocyte ratio, NLR=Neutrophil-to-lymphocyte ratio, PLR=Platelet-to-lymphocyte ratio, PNR=Platelet-to-neutrophil ratio, COP=Cyclophosphamide, vincristine, and prednisolone, L-COP=Cyclophosphamide, vincristine, and prednisolone with L-asparaginase

**Table-6:** Cut-off values determined by ROC curve and AUC analysis for the significant hematological parameters for overall survival at 180 days and 365 days.

Parameters	180 days					365 days				
	
	Cut-off	p-value	AUC	Sens.	Sp.	Cut-off	p-value	AUC	Sens.	Sp.
COP protocol										
Monocyte0 (×10^3^/uL)	0.637	0.078	0.250	0.500	0.400	0.637	0.577	0.405	0.571	0.500
Creatinine0 (mg/dL)	0.65	0.887	0.472	0.889	0.500	0.65	0.719	0.438	0.875	0.429

**Parameters**	**180 days**					**365 days**				
	
	**Cut-off**	**p-value**	**AUC**	**Sens.**	**Sp.**	**Cut-off**	**p-value**	**AUC**	**Sens.**	**Sp.**

L-COP protocol										
Albumin0 (g/dL)	2.45	0.217	0.648	0.867	0.545	2.45	0.704	0.549	0.714	0.368
Albumin4 (g/dL)	2.50	0.722	0.545	0.909	0.263	2.50	0.962	0.507	1.000	0.222
TWBC4 (×10^3^/uL)	16.255	0.716	0.545	0.167	0.818	10.675	0.853	0.526	0.500	0.737

AUC=Area under the curve, Sens.=Sensitivity, Sp.=Specificity, Albumin0=Pre-treatment serum albumin concentration, Albumin4=Post-treatment serum albumin concentration at 4 weeks, TWBC4=Post-treatment total white blood cell count at 4 weeks, Monocyte0=Pre-treatment monocyte count, Creatinine0=Pre-treatment serum creatinine concentration, COP=Cyclophosphamide, vincristine, and prednisolone, L-COP=Cyclophosphamide, vincristine, and prednisolone with L-asparaginase

Conversely, the cutoff values of those parameters affecting overall survival at 180 and 365 days in dogs with multicentric lymphoma treated with COP or L-COP could not be established owing to the loss of significance in the ROC curve and AUC analysis (Table-6).

## Discussion

This study investigated the effect of hematological and blood chemistry parameters pre-treatment and 4 weeks post-treatment on the survival outcomes of dogs with multicentric lymphoma treated with either the COP or L-COP protocols. In dogs with multicentric lymphoma treated using the COP protocol, pre-treatment monocytosis or azotemia was correlated with poor survival outcomes at 180 and 365 days. These dogs also had significantly shorter MST than dogs with normal monocyte counts or serum creatinine concentrations. In contrast, hypoalbuminemia was a strong negative prognostic indicator of survival outcomes in dogs with multicentric lymphoma treated with the L-COP protocol. Dogs with hypoalbuminemia pre-treatment or 4 weeks post-treatment had a significantly shorter ST than those with a normal serum albumin concentration. In addition, dogs with multicentric lymphoma and leukocytosis treated with the L-COP protocol at 4 weeks post-treatment had a significantly shorter MST than dogs with a normal white blood cell count. However, this study did not aim to compare the treatment outcomes between the two protocols.

Pre-treatment serum albumin concentration has been used as a prognostic indicator for various neoplastic diseases such as non-small cell lung cancer [19], colorectal carcinoma [20], acute amyloid leukemia [21], and lymphoma [22–24] in humans. Previous studies have revealed that hypoalbuminemia has a potential effect on survival outcomes in patients with this disease owing to an increased risk of chemotherapy-induced toxicity, response to systemic inflammation, poor nutritional status, and suboptimal response to chemotherapy [19-24]. In contrast, few studies have shown an association between the pre-treatment serum albumin concentration and survival outcomes in canine lymphoma [14, 15]. The current results are consistent with those of previous studies by Dank *et al*. [14] and Fontaine *et al*. [15], in that a significant influence of pre-treatment hypoalbuminemia on overall survival was detected in dogs with multicentric lymphoma treated with CHOP-based protocols. This study also found that post-treatment serum albumin at 4 weeks strongly affected overall survival in dogs with multicentric lymphoma treated with the L-COP protocol. Although this study could not establish the cutoff value of serum albumin concentration for prognostic survival outcomes, the result was similar to that of a previous study by Dank *et al*. [14] in canine hepatic lymphoma, in which dogs with serum albumin concentration <2.5 mg/dL were 6.2 times more likely to have a shorter ST than those with a normal serum albumin concentration. Interestingly, 8 out of 26 (30.77%) dogs treated with the L-COP protocol presented with hypoalbuminemia at pre-treatment. Unfortunately, one dog died after 16 days after treatment. Four weeks post-treatment, 4 out of 24 (16.67%) dogs presented with hypoalbuminemia. Among the four dogs, one dog had hypoalbuminemia and three had a normal pre-treatment serum albumin concentration. It should be considered that hypoalbuminemia at 4 weeks post-treatment may also affect the survival outcomes in dogs with multicentric lymphoma treated with the L-COP protocol. In addition, the current study was unable to determine the effect of hypoalbuminemia on survival outcomes in dogs treated with the COP protocol. This was because none of the dogs in this group presented with hypoalbuminemia pre-treatment and 4 weeks post-treatment.

Serum albumin plays an important role in chemotherapy. It acts as a natural circulating carrier for chemotherapeutic drugs such as cyclophosphamide and is involved in drug degradation and clearance [25]. Previous studies by Wang et al. [19] and Kaneko *et al*. [22] have shown that neoplastic patients with hypoalbuminemia were less responsive to chemotherapy and developed more chemotherapy-induced toxic symptoms. Moreover, hypoalbuminemia indicates the nutritional status, which is a crucial factor in cancer patients treated with chemotherapy. Studies have revealed that pre-treatment hypoalbuminemia is associated with tumor progression, poor survival outcomes, and intolerance to intensive chemotherapy [23, 26]. Furthermore, post-treatment hypoalbuminemia resulting from malnutrition is also regarded as a negative prognostic factor for human cancer [27, 28]. In general, post-treatment hypoalbuminemia can be caused by decreased protein intake, decreased production in the liver due to hepatic failure or response to systemic inflammation, or increased loss of albumin, such as in protein-losing enteropathy and nephropathy [29]. The possible mechanisms of pre-treatment hypoalbuminemia in canine lymphoma have been described as a decrease in albumin production owing to the response to systemic inflammation associated with tumor progression [14, 19, 22, 24]. Second, chronic anorexia or starvation in malignant conditions leads to poor nutrition status, including hypoalbuminemia [15, 30]. Finally, hypoalbuminemia can be caused by protein-losing nephropathy owing to impaired glomerular permselectivity after lymphoma [31]. Although the causes of hypoalbuminemia in this study could not be proven because of a lack of information about urinalysis and advanced liver investigations, such as cytological or histopathological examinations, none of the enrolled dogs with hypoalbuminemia presented with azotemia. In addition, 4 out of 8 dogs (50%) with hypoalbuminemia presented with high concentrations of ALT and ALP, with no clinical signs related to liver dysfunction. Since most dogs with hypoalbuminemia in this study had poor condition scores (3 out of 9), malnourishment was likely the contributing factor. Regarding protein-losing enteropathy causing hypoalbuminemia, none of the dogs in this study presented with gastrointestinal signs, particularly chronic diarrhea. Therefore, protein-losing enteropathy is unlikely to cause hypoalbuminemia.

In addition to albumin, this study found that leukocytosis at 4 weeks post-treatment affected the survival outcomes in dogs treated with the L-COP protocol. Previous studies by Gavazza *et al*. [9] and Dank *et al*. [14] have similarly shown that leukocytosis impairs the complete response rate and ST in dogs with lymphoma. This abnormality may be related to stress or inflammation due to lymphoma infiltration and is associated with poorer outcomes [14]. Considering the dogs treated with the COP protocol, dogs with pre-treatment monocytosis had poor overall survival at 180 days. The role of monocytes in lymphoma development may explain this phenomenon. Monocytes support the survival and proliferation of neoplastic B-cells and suppress the proliferation of normal T cells. Furthermore, they contribute to the suppression of host antitumor immunity and play a crucial role in tumor angiogenesis, thereby promoting tumor growth [12]. Previous studies by Marconato *et al*. [12] and Tadmor *et al*. [32] have shown that monocytosis is a negative indicator of survival outcomes in humans and dogs with lymphoma. In this study, the hematological results of all dogs were investigated using an automated hematology analyzer. However, in dogs presenting with leukocytosis, peripheral blood smears are performed to confirm the presence of lymphoblasts in stage V of lymphomas. Previous studies by Xiang *et al*. [33] and Thongsahuan *et al*. [34] have shown that an automated hematological analyzer provides a good-to-excellent correlation for evaluating white blood cells compared to that obtained through manual counting. However, a manual count should be performed to accurately distinguish between true monocytosis and relative monocytosis caused by an increase of lymphoblasts in stage V of lymphoma [33, 34].

In this study, dogs displaying pre-treatment azotemia, as indicated by an increase in creatinine concentration, had a higher risk of dying within 180 and 365 days, along with a shorter MST. Although an increased BUN concentration reflects decreased glomerular filtration, other factors, such as a high-protein diet and small bowel hemorrhage, can also increase BUN levels. Conversely, BUN levels can decrease because of hepatic failure and a low-protein diet [35]. Creatinine is an indirect indicator of kidney function because it directly reflects a decrease in the glomerular filtration rate, and it is more reliable than BUN because it is not affected by a high-protein diet [35]. However, serum creatinine is derived from the degradation of creatinine and creatinine phosphate, which are primarily found in muscle tissue. Dogs with muscle loss, particularly malnourished dogs, may have lower serum creatinine concentration [36]. Moreover, pre-treatment azotemia can be caused by decreased renal perfusion due to dehydration [35]. This may have been caused by decreased water intake owing to anorexia in dogs with multicentric lymphoma. Numerous drugs, including chemotherapeutic drugs, are metabolized by the liver, and eliminated by the kidneys. Dogs presenting with azotemia tend to have decreased drug clearance and an increased risk of chemotherapy-induced toxicity [37, 38]. Considering the effect of the L-COP protocol on kidney function, while cyclophosphamide has mostly been reported to be related to hemorrhagic cystitis [39], few studies have reported drug-related nephrotoxicity of cyclophosphamide [40, 41]. Although limited information is available, L-asparaginase and vincristine have direct effects on kidney function [42, 43]. In contrast, prednisolone has anti-inflammatory effects that improve kidney function and hemodynamic stability [44, 45]. In addition, azotemia has been reported to affect the survival outcomes of human lymphoma [46].

A high ALP concentration pre-treatment and 4 weeks post-treatment was observed in 13 dogs treated with the COP or L-COP protocols in this study. Eight of the 13 (61/54%) dogs with high ALP levels underwent diagnostic imaging, such as abdominal radiography and/or ultrasonography, which indicated that these dogs had hepatomegaly and altered hepatic parenchyma. Owing to the limitations of the retrospective study model, the causes of these abnormalities could not be investigated. Increased ALP concentration is one of the most frequently observed biochemical abnormalities in dogs and has a high sensitivity but low specificity for hepatobiliary diseases in dogs owing to the presence of several isoenzymes [47]. The possible causes of the increase in ALP concentration in this study were inflammation, neoplasia, cholestasis, or endogenous corticosteroid-induced ALP, and exogenous corticosteroid-induced ALP due to steroid administration in the chemotherapeutic protocol [47, 48]. However, our study showed that increased ALP concentrations did not affect survival outcomes, which is consistent with a previous study by Wiedemann *et al*. [49], which revealed that ALP did not influence survival outcomes in canine lymphoma. Finally, an increased pre-treatment ALT concentration was observed in five dogs treated with the COP protocol, and 3 of these 5 (60%) dogs had hepatomegaly. Although the final diagnosis of this event could not be established, hepatic parenchymal infiltration may have been the cause [1].

Several hematological and blood chemistry parameters have been identified to influence survival outcomes, including anemia or thrombocytopenia commonly associated with bone marrow involvement in stage V lymphoma [11, 13, 50], chronic inflammatory leukogram [9], LMR value >1.2 [12], neutrophilia [13], and higher serum globulin [13]; however, in this study, there was no evidence of any abnormalities significantly associated with survival outcomes.

## Limitations

This study had certain limitations. First, the selection of chemotherapeutic protocols and dosage could not be controlled and depended on the oncologist’s decision, including the further investigation of the hepatic parenchyma or urinalysis using a retrospective model. Second, as this was a retrospective study, stage IV lymphoma was mainly classified based on the ultrasonographic appearance of hepatic and splenic involvement. While cytology or histopathology is the gold standard for diagnosing hepatic and splenic involvement in this disease, previous studies have shown that the ultrasonographic appearances of the spleen and liver, particularly a leopard-spotted splenic parenchyma, provide high specificity in detecting lymphomatous infiltration [51, 52]. Third, all dogs were diagnosed with multicentric lymphoma based on cytological results, with most of them classified as having high-grade lymphoma according to the Kiel cytological classification. This study revealed the effect of hypoalbuminemia on the survival outcomes in dogs with high-grade multicentric lymphoma treated using the L-COP protocol. Previous studies by Valli *et al*. [5] and Bennett *et al*. [6] have shown that the immunophenotype (B- or T-cell origin) and histological classification type affect survival outcomes in dogs with multicentric lymphoma. However, owing to the retrospective nature of the current study, there is a lack of information regarding the WHO histologic classification type, which may have led to selection bias and influenced the survival outcomes in this study. Further studies should investigate the correlation between hypoalbuminemia and survival outcomes, as well as the WHO histological classification, including low-grade lymphoma. Fourth, the final diagnoses of the causes of monocytosis and azotemia were not established because of the limitations of the retrospective nature of this study. These abnormalities may be influenced by lymphoma and other concurrent diseases, which can interfere with the study results. Nevertheless, the study demonstrated that dogs with multicentric lymphoma, monocytosis, or azotemia had a shorter ST than dogs with normal monocyte count and creatinine levels. Fifth, the statistical power of the analysis may have been affected by the small number of samples. Finally, none of the dogs treated with the COP protocol presented with hypoalbuminemia. Therefore, we could not determine the effect of hypoalbuminemia on the survival outcomes of the dogs in this group.

## Conclusion

This study revealed that pre-treatment hypoalbuminemia had a significant effect on overall survival at 180 days in dogs with high-grade multicentric lymphoma treated with the L-COP protocol. Moreover, post-treatment hypoalbuminemia affected survival outcomes at 180 and 365 days. Dogs with hypoalbuminemia tended to have shorter MST than those with normal serum albumin concentrations. Therefore, hypoalbuminemia could be used as a simple negative prognostic indicator in dogs with high-grade multicentric lymphoma treated with the L-COP protocol. In this study, other abnormalities affecting survival outcomes were leukocytosis, monocytosis, and azotemia.

### Data availability

The data used to support the findings of this study are available from the corresponding author upon request.

## Authors’ Contributions

NC, AR, CT, and SS: Contributed to the conception and design of the study. PS, PT, SSi, and SS: Performed sample and data collection. NC, AR, and SS: Performed data validation and statistical analysis. NC and SS: Drafted the manuscript and prepared the graphs. NC, AR, and CT: Supervised the study. All authors have read, reviewed, and approved the final manuscript.
